# Editorial: Resilience processes and children's development within socio-ecological contexts

**DOI:** 10.3389/fpsyg.2024.1429178

**Published:** 2024-05-29

**Authors:** Shannon Tierney Lipscomb, Kyong-Ah Kwon, Philip Jefferies, Francesca Giordano

**Affiliations:** ^1^College of Health, Oregon State University-Cascades, Bend, OR, United States; ^2^College of Education, University of Oklahoma, Norman, OK, United States; ^3^Resilience Research Centre, Faculty of Health, Dalhousie University, Halifax, NS, Canada; ^4^Department of Psychology, Faculty of Humanities, University of Johannesburg, Johannesburg, South Africa; ^5^Department of Psychology, Catholic University of the Sacred Heart, Milan, Lombardia, Italy

**Keywords:** resilience, child, youth, context, family, community, early care and education

Children and youth experience adversity that impacts their development, relationships, learning, and wellbeing. Resilience can mitigate these impacts. This Research Topic of articles advances the science of resilience by uncovering new evidence about how resilience develops as a dynamic interplay between children and their socio-ecological contexts, such as family, school, neighborhood, and culture. Consistent with leading experts (e.g., Masten, 2018; Ungar, 2021), authors conceptualize resilience as a process of adaptation in the context of adversity, which draws upon internal and external resources. Studies examine these processes from infancy (Chazan-Cohen et al.; Horm et al.) and preschool (Clark et al.; Liu et al.; Merculief et al.) to middle childhood (Bates et al.) and adolescence (Giordano et al.; Han et al.).

## Resilience processes unfold within social-ecological contexts

### Adults as mediators of children's resilience

Development, particularly in early childhood, is interdependent. Yet, the nuanced pathways through which adults nurture children's resilience are not yet well understood. This Research Topic contributes significant advances by delving into the complexities of such processes. For example, although parental warmth has been clearly linked to child wellbeing for decades, by applying a resilience framework, Bates et al. also documented a unique protective effect of maternal warmth in attenuating the deleterious effect of prenatal substance use on children's prosocial behavior when children lived with biological (rather than adoptive) parents.

Two articles uncovered links between adult caregivers' post-traumatic growth and young children's development. Clark et al. documented that early childhood educators' post-traumatic growth was associated with fewer difficulties in preschoolers' executive functioning and metacognition. While much is still to be learned about mechanisms underlying these associations, this work expands evidence of the importance of educator wellness to children's development (e.g., Jeon et al., 2019; Kwon et al., 2019) to encompass trauma and resilience. Liu et al. identified both promotive and protective effects of parents' post-traumatic growth to mitigate parental distress in the context of material hardship during the COVID-19 pandemic, which in turn attenuated preschoolers' social and behavioral challenges. This article provides a foundation for examining how parents' growth-oriented responses to stressors can set in motion a chain of experiences within families that support resilience. Such an approach is akin to the well-documented family stress model (Masarik and Conger, 2017), with a focus on resilience.

Collectively, these studies highlight the importance of supporting adults' resilience within strategies to nurture resilience with children. Chazan-Cohen et al. directly examine one such approach for families with young children and low-income. Findings revealed that directly supporting parents with job-related education and training contributed to more supportive home environments and children's outcomes. Supporting parental self-development, not only training parents in adult-child interaction, may be an important avenue to foster resilience of both parents and children facing adverse situations such as poverty. Finally, the study by Horm et al. reminds us that exchanges between adults and children that nurture resilience are shaped not only by adults but also by children's own behavior, such as the self-regulation of infants/toddlers within a classroom.

### Cultural and community supports for children's resilience

This Research Topic of articles advances understanding of unique cultural and/or community aspects of resilience through representation of racial, ethnic, and cultural groups (American Indian and Alaska Native; Black, Latino/Latinx, and White), and countries (e.g., China, Ukraine, and the U.S.). In particular, Liu et al. found that Black and Latina mothers showed more post-traumatic growth than white mothers during the COVID-19 pandemic, which the authors interpret as potentially reflecting both cultural differences in closer social networks, and more prior experience with adversity, specifically discrimination, upon which to draw strength.

Studies also underscore how cultural experiences can foster resilience of children and youth. For instance, among American Indian and Alaska Native preschool children, language, and cultural socialization activities that strengthen spiritual and cultural connections, served as a resilience factor by promoting children's executive function (Merculief et al.). In a qualitative study Giordano et al. identified community connections as protective processes, and documented cultural nuances in how Ukrainian youth experience resilience in the context of war and displacement.

## Methodological approaches to studying the complexities of resilience

Studies in this Research Topic illustrate the importance of exploring resilience within appropriate theory and methods to manage complexity. In particular, several articles illustrate analytic methods for examining resilience processes, including both quantitative (e.g., mediation, moderation) and qualitative (e.g., thematic analysis and individual differences) approaches. Chazan-Cohen et al., Han et al., Horm et al., and Liu et al. draw on mediation analyses. For example, Liu et al. examined how the emotional distress of parents may mediate relationships such as household financial difficulties on child behavior. Moderation analyses were employed by Bates et al. to investigate whether effects of neighborhood safety and financial security on prosocial behavior varied depending on whether a child was raised in a household with their biological or adoptive parents. Both moderation and mediation are important to deepen understanding of resilience processes. Mediation examines mechanisms, while moderation helps to identify what works for whom, and in what contexts (Ungar, 2011). Leveraging strengths of a qualitative perspective, the thematic analysis of transcripts from Ukrainian youth engaged in a resilience training intervention during a time of war allowed for identification of cultural and contextual nuance, as well as individual differences (Giordano et al.).

## Conclusion

The articles in this Research Topic represent important advancements in the study of resilience as a dynamic process among children and the complex environments they navigate. These studies establish foundations for future research about both supporting the adults in children's lives and attending to cultural resources within efforts to build children's resilience. Findings have implications for intervention to strengthen resilience, such as by informing tailored interventions based on moderation or subgroup effects (e.g., Bates et al.), embedding interventions in cultural contexts (e.g., Merculief et al.), and providing flexibility to meet both individual and culturally-specific needs (Giordano et al.).

Additionally, these articles bring forth emerging challenges with which the field must grapple. Since a vast array of developmental processes and experiences can be examined as *resilience*, we must ask when research is truly studying resilience rather than specific constructs that may be implicated in resilience processes but do not actually constitute resilience themselves. Consistent with recommendations by Ungar (2019), our approach with this Research Topic of articles was to include studies that examined processes (rather than traits), measured adversity, and explained outcomes. Advancement of science to move the needle on implementation will require researchers to delve more deeply into interpersonal and developmental processes across contexts. For example, two studies (Clark et al.; Liu et al.) in this Research Topic examined post-traumatic growth. Future research must uncover the sources of such growth and processes that foster healing. By homing in on specific processes while also aggregating evidence across broad themes, resilience science holds great promise to effectively inform strategies to help children and youth thrive.

## Author contributions

SL: Conceptualization, Writing—original draft, Writing—review & editing. K-AK: Conceptualization, Writing—original draft, Writing—review & editing. PJ: Conceptualization, Writing—original draft, Writing—review & editing. FG: Conceptualization, Writing—original draft, Writing—review & editing.
